# Breaking the barriers: the role of gut homeostasis in Metabolic-Associated Steatotic Liver Disease (MASLD)

**DOI:** 10.1080/19490976.2024.2331460

**Published:** 2024-03-21

**Authors:** Raquel Benedé-Ubieto, Francisco Javier Cubero, Yulia A. Nevzorova

**Affiliations:** a Department of Immunology, Ophthalmology and ENT, Complutense University School of Medicine, Madrid, Spain; b Centro de Investigación Biomédica en Red de Enfermedades Hepáticas y Digestivas (CIBEREHD), Madrid, Spain; c Instituto de Investigación Sanitaria Gregorio Marañón (IiSGM), Madrid, Spain

**Keywords:** MASLD, microbiota, gut-liver axis, gut barriers, FMT, therapies

## Abstract

Obesity, insulin resistance (IR), and the gut microbiome intricately interplay in Metabolic-associated Steatotic Liver Disease (MASLD), previously known as Non-Alcoholic Fatty Liver Disease (NAFLD), a growing health concern. The complex progression of MASLD extends beyond the liver, driven by “gut-liver axis,” where diet, genetics, and gut-liver interactions influence disease development. The pathophysiology of MASLD involves excessive liver fat accumulation, hepatocyte dysfunction, inflammation, and fibrosis, with subsequent risk of hepatocellular carcinoma (HCC). The gut, a tripartite barrier, with mechanical, immune, and microbial components, engages in a constant communication with the liver. Recent evidence links dysbiosis and disrupted barriers to systemic inflammation and disease progression. Toll-like receptors (TLRs) mediate immunological crosstalk between the gut and liver, recognizing microbial structures and triggering immune responses. The “multiple hit model” of MASLD development involves factors like fat accumulation, insulin resistance, gut dysbiosis, and genetics/environmental elements disrupting the gut-liver axis, leading to impaired intestinal barrier function and increased gut permeability. Clinical management strategies encompass dietary interventions, physical exercise, pharmacotherapy targeting bile acid (BA) metabolism, and microbiome modulation approaches through prebiotics, probiotics, symbiotics, and fecal microbiota transplantation (FMT). This review underscores the complex interactions between diet, metabolism, microbiome, and their impact on MASLD pathophysiology and therapeutic prospects.

## Introduction

By the beginning of the third millennium, the prevalence of obesity and metabolic diseases dramatically increased, and became a real burden for health systems in the Western world.^1^

Metabolic dysfunction-associated steatotic liver disease (MASLD), previously known as Non-Alcoholic Fatty Liver disease (NAFLD), is a global disease that affects about 25% of the world population.^2^ Nowadays, the overall prevalence of MASLD is growing in parallel with the worldwide epidemic of obesity. The sedentary lifestyle, lack of physical exercise, an hypercaloric diet mainly composed by fat, refined sugars and carbohydrates are the main reasons and risk factors for the development of MASLD.^3^ However other risk factors, as gender, genetic predisposition, age, smoking, or drug and alcohol consumption can influence on MASLD progression and make its pathophysiology more heterogeneous and complex.^4^

Although the liver was initially assessed as the main organ involved in the development of MASLD, nowadays it became clear that a complex crosstalk between several organs involved in the progression of MASLD. In fact, MASLD is rather a systemic disease where obesity, metabolic syndrome (MS), white adipose tissue (WAT) inflammation and fatty liver (FL) tightly interact and push each other to further pathophysiological stages of such as hepatic inflammation, fibrosis, cirrhosis and hepatocellular carcinoma (HCC).^5^

Recently, a growing body of experimental and clinical evidence indicated that central aspects of liver function also strongly depend on the coordinated action of gut. In fact, anatomical cellular and molecular paths connect both organs and synchronize their work. The intimate connection and the strict mutual cooperation between the gut and the liver realizes a functional entity called “gut-liver axis”.^6^

This connection involves reciprocal interactions at cellular and molecular levels between the gut, its microbiota, and the liver, which are influenced by various factors such as the diet, genetics, and the environment.^7^

MASLD is associated with changes in intestinal microbiota as well as intestinal barrier integrity suggesting an important role of the gut-liver axis in the development of disease.^8^ The effect of obesity, insulin resistance (IR), the interaction and metabolic crosstalk between the gut and the gut microbiome on MASLD will be discussed in this review.

## Hepatic features of MASLD

MASLD is characterized by an excessive fat accumulation (>5%) in the liver.^9,10^

The increased uptake of free fatty acids (FFA) and lipogenesis, defects in FFA oxidation, and decreased lipids export all contribute to the impaired hepatic lipid metabolism.^11^ Extensive fat accumulation in the hepatic parenchyma led to excessive mitochondrial reactive oxygen species (ROS) production. Oxidative stress can induce mitochondrial dysfunction, and hepatocyte cytotoxicity, ending in cell death.^12^ Extensive hepatocyte cell death and liver damage induce the recruitment of immune cells and the activation of proinflammatory pathways that further increase liver damage and induce the over presence of proinflammatory cytokines (TNFα, TGFβ, IL6, IL1β) that consequently activate the hepatic stellate cells (HSCs).^13^ These events trigger the production of extracellular matrix (ECM) inducing collagen deposition and leading in 1–2% of the cases to advanced stages of fibrosis and cirrhosis. Advanced liver fibrosis and cirrhosis are the major risk factors for HCC.^14,15^

## Gut-liver axis

The gut is one of the most extensive mucosal surfaces in the human body and serves as a barrier that safeguards against pathogenic microorganisms and toxic substances. The gastrointestinal (GI) system is also responsible for the digestion and absorption of food.^16^ The gut and the liver are in constant crosstalk due to anatomical and functional interactions. The gut is the initial organ that receives the nutrients from the food. Consequently, the liver is exposed to substances that come directly from the gut via portal vein supplementation.^16,17^

**Figure 1.**
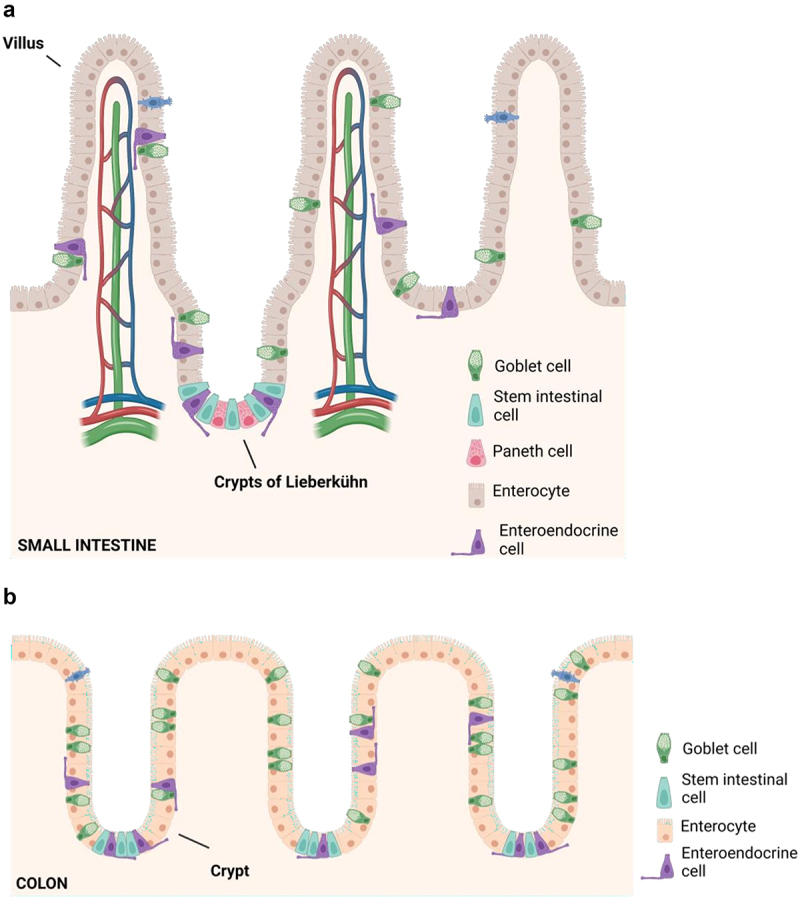
A. Epithelium organization of the small intestine. Graphical representation of villi, crypts and different type of intestinal cells. B. Colon epithelium. Graphical representation of crypts and different type of colonic cells. Figure created with BioRender.com.

The gut is divided into two parts, the small intestine that is also segmented into the duodenum, jejunum and ileum and the large intestine that is formed mainly by the colon and the rectum.^6^ The structure of the small intestine is characterized by the presence of villi and crypts that increase the surface of absorption. Its internal structure is complex, and substances can be absorbed by active or passive transport^18^ (Figure 1a).

The colon lacks villi and displays a flatter surface. Epithelial cells are continuously renewed from invaginations known as the crypts of Lieberkühn, where multipotent stem cells give rise to the different cell types of intestinal epithelium: columnar absorptive cells or enterocytes, mucous secreting goblet cells, enteroendocrine cells and Paneth cells^6,19,20^ (Figure 1b).

The gut and the liver are anatomically linked through portal circulation which is the physical connection of the gut-liver axis. However, the presence of the intestinal barrier restricts the extent to which the intestine and liver are connected.^6^

The gut is semi-permeable, it allows the passage of some substance while others remain in the GI tract.^21^ Hence, GI tract is not only responsible for digestion and absorption of nutrients and other substances essential for life, but it also constitutes the primary defense against pathogens and hazardous substances preventing them to reach the blood, liver, spleen, and other organs.^22^

However, some environmental factors, changes in the gut microbiome, toxins and substances like the alcohol or drugs, excess of fat and intestinal inflammation itself can induce changes in the enterocytes and in the mucosa, and consequently increase the intestinal permeability also called – leaky gut.^16^

## The gut barriers

**Figure 2.**
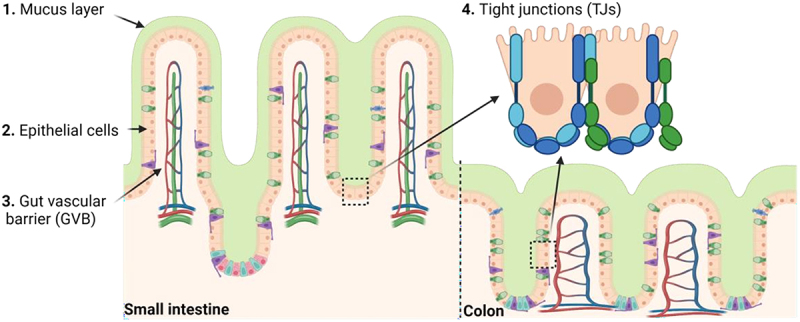
Gut mechanical barrier components. 1. Mucus layer is the outer mechanical barrier composed by mucus. 2. Epithelial cells: enterocytes, goblet cells, enteroendocrine cells, Paneth cells and microfold cells. 3. Tight junctions (TJs) are composed of proteins that control the paracellular pathway, as well as adherent junctions, desmosomes, and gap junctions. 4. Gut vascular barrier (GVB) constitutes the inner layer of defense, and it is composed by endothelial cells linked by TJs among others and different proteins that play a fundamental role regulating blood vessel permeability. Figure created with BioRender.com.

The gut barrier is comprised of three major lines of defense: 1. The mechanical barrier; 2. The immune barrier; and 3. The biological barrier. These barriers can interact with each other to maintain gut homeostasis^23^ (Figure 2).

### The mechanical barrier

One of the components of the gut barrier is the mechanical layer composed by: a) The intestinal epithelial cells (IEC); b) Goblet cells; c). Paneth cells, d) Glial cells and e) Mucus layer.^22,24,25^ All layers offer protection against mechanical, chemical, and biological agents.^26^ All cells of the gut barrier are replenished by a group of stem cells located in the intestinal crypts.^23^

#### The mucus layer

**Figure 3.**
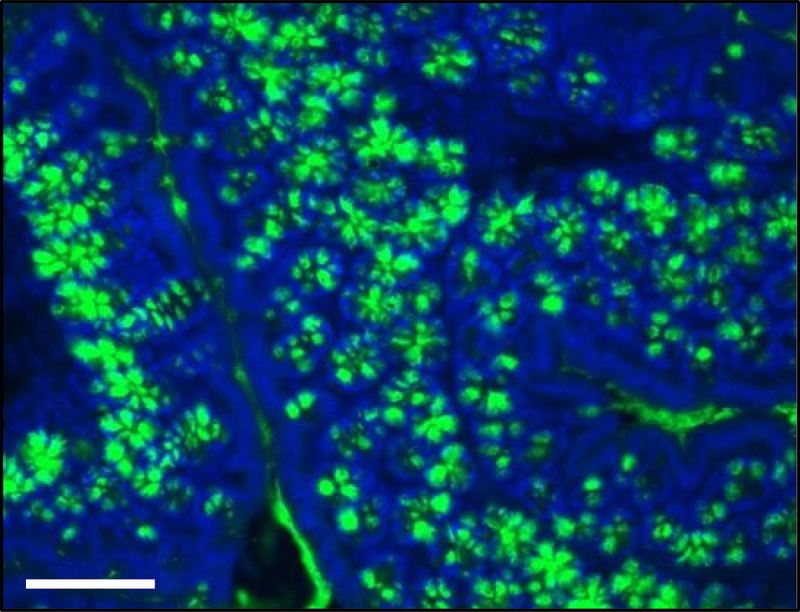
Photomicrograph of immunofluorescence (IF) preparation of colon stained with mucin-2 antibody. Nuclei are stained in blue. Staining was performed in 5 μm sections from paraffin embedded colon from a C57Bl/6J mouse 20 weeks age fed with chow diet. Scale = 100 μm.

The mucosal surface of the GI tract is covered by mucus a substance secreted by goblet cells. The major building blocks and critical structural component of the mucosal barrier are mucins, which are large, highly glycosylated proteins giving the mucus its gel-like properties. The mucus produced by the Goblet cells is the classical gel-forming mucins (MUC), MUC2 (Figure 3), MUC5AC, MUC6 and MUC5B which are secreted by the intestine, stomach surface, stomach glands and salivary glands, respectively.^27^

The main function of the mucus layer is to shield the intestinal cells from external agents and to facilitate nutrient absorption.^27^ The stomach and the colon have double layer of mucus, while the small intestine has only a single layer.^28^

n the stomach and first part of the duodenum the mucus is thicker as it serves as a stable, non-stirred layer that supports surface neutralization of acid and maintains a pH gradient from acidic to nearly neutral at the mucosal surface. Furthermore, the mucus layer acts as a physical barrier, preventing luminal pepsin from reaching the underlying mucosal surface.^29^

In the colon there are two layers of mucus. The inner layer is densely packed, firmly attached to the epithelium and is impenetrable to bacteria. When the inner layer is penetrable to bacteria, they reach the epithelial cells and trigger inflammation. The outer layer is movable, has an expanded volume and is the natural habitat for the commensal bacteria.^28^ Due to proteolytic cleavages of MUC2 and increased pore sizes bacteria into the mucin net-like structure and gain access to the plentiful mucin-bound carbohydrates that can be utilized by the bacteria as an energy source. In turn, the commensal bacteria produce a variety of metabolites, some of which are useful to the host.^27^

#### The epithelial cells

The gut epithelium is not permeable to hydrophilic solutes, which means that molecules and nutrients can only pass through it via specific transporters. There are two primary pathways for transport: the transcellular route, which includes aqueous pores, active carrier-mediated absorption for nutrients and endocytosis, and the paracellular route, which allows ions and hydrophilic molecules to pass through.^30^

#### Protein junctional complexes

**Figure 4.**
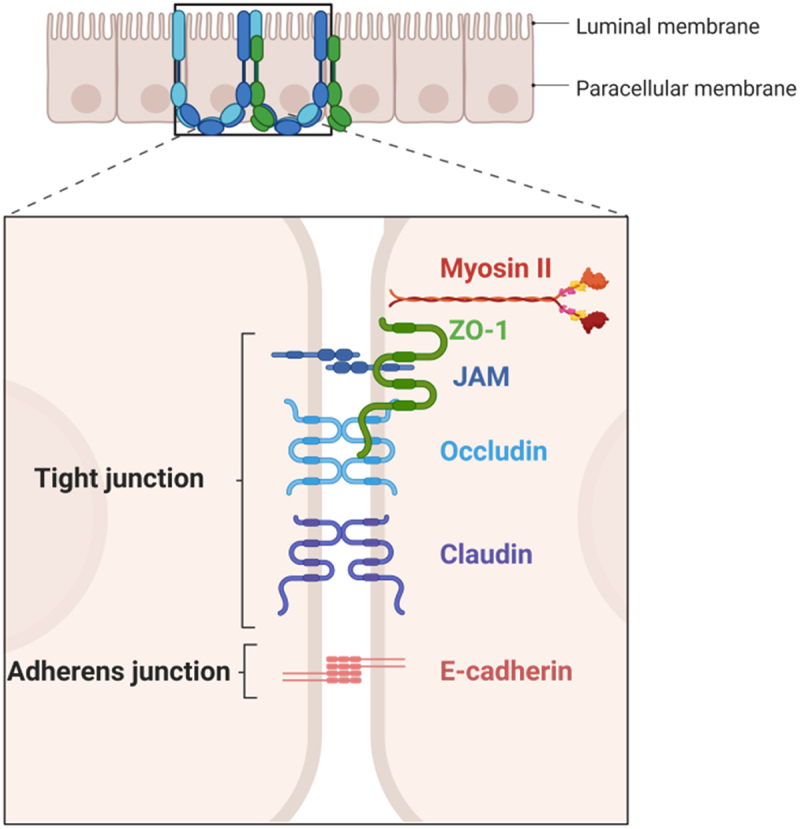
Schematic representation of TJ in gut. The paracellular pathway is controlled by a group of proteins known as junctional complexes that include, adherent junctions, gap junctions and tight junctions (composed by transmembrane proteins as claudins, occludin and junctional adhesion molecules (JAM)) which interact with the zona occludens (ZO) family of scaffolding proteins and the cytoskeletal actomyosin ring. Figure created with BioRender.com.

**Figure 5.**
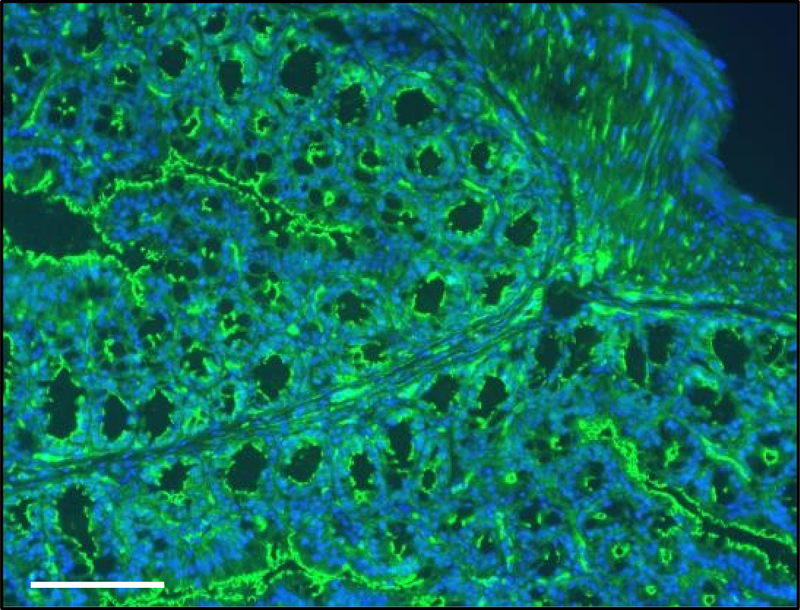
Photomicrograph of immunofluorescence (IF) preparation of colon stained with ZO-1. Nuclei are stained in blue. Staining was performed in 5 μm sections from paraffin embedded colon from a C57Bl/6J mouse 20 weeks age fed with chow diet. mounting. Scale = 100 μm.

The paracellular pathway is controlled by a group of proteins known as junctional complexes that include tight junctions (TJs), adherent junctions, desmosomes and gap junctions. They are located at the apical ends of the lateral membranes of IECs. They are composed by transmembrane proteins, occludins, claudins, junctional adhesion molecules and tricellulins,^31,32^ the Marvel domain-containing proteins and immunoglobulin superfamily, which interact with the cytoskeletal actomyosin ring.^33^ The cytosolic scaffold proteins, such as zona occludens (ZO) and claudin proteins interact among them and anchor the transmembrane proteins to the actin cytoskeleton. This interaction is vital to maintain TJ barrier integrity and minimize the gut permeability^34^ Figure 4. Representative picture of ZO-1 in colon is shown in Figure 5.

Studies on gut permeability reveal that TJs selectively enable the passage of substances across the gut barrier. This selectivity is achieved through the existence of two distinct classes of trans-TJ flux pathways, each with varying size and charge selectivity. These pathways can also be differentiated based on their capacity. The pore pathway has the ability to transport significant amounts of small, uncharged solutes and specific ions, whereas the leak pathway permits only small quantities of larger molecules and ions regardless of charge to pass.^35^ As a result, TJs provide a mechanical boundary between the luminal space and other components of the intestinal barrier.^36^

#### The gut vascular barrier

The gut vascular barrier (GVB) represents the inner layer of defense in the multi-layered intestinal barrier system that finely regulates the translocation of substances from the intestinal lumen to the systemic circulation.^37^

The GVB is mainly composed by gut endothelial cells and pericytes, which are linked by adherent junctions, TJs, catenin and cadherin proteins, and play a vital role in regulating the permeability of blood vessels.^38^ These cells are fenestrated. The small pores are delimited by a fenestrae diaphragm regulated by plasmalemma vesicle-associated protein-1 (PV-1), which is essential for maintaining endothelial homeostasis and permeability.^39^ Moreover, the defective GVB has been associated to an increased expression of PV-1.^38,40,41^

In addition to their role in regulating vascular permeability, endothelial cells also play a role in mucosal immunology, expressing Toll-like receptors (TLRs) and adhesion molecules such as E-selectin, vascular cell adhesion molecule-2 (VCAM-1), and intercellular adhesion molecule-1 (ICAM-1). They form a layer with pericytes and enteric glial cells underneath the IEC called the GVB. Enteric glial cells help to maintain the integrity of the intestinal barrier by communicating with enteric neurons and releasing soluble factors such as S-nitroso glutathione, which controls paracellular permeability by increasing TJ protein expression.^39,42^

### The immune barrier

The immune barrier is composed by a diverse range of immune cells and other types of cells that are part from the intestinal epithelium that exert immune functions.^43^

The epithelium layer is the first line of defense against pathogens. IECs have fundamental immune-regulatory functions. They promote the development and differentiation of immune-regulatory CD8αα intraepithelial lymphocytes through the trans-presentation of IL15. Moreover, the overexpression of IL15 is crucial for facilitation the movement and localization of protective γδ-intraepithelial lymphocytes within the small intestine’s epithelial lining.^44^

Furthermore, IECs exhibit the expression of anti-inflammatory cytokines like IL10, which likely contributes to fostering tolerance toward commensal bacteria maintaining the integrity of the epithelium.^45^

IECs recognize pathogenic molecule patterns where MyD88 is a central adaptor molecule involved. Additionally, IECs are also involved in the adaptive immune regulation of gut homeostasis mediated by secretory immunoglobulin A (SIgA).^46^

Specialized IECs such as Goblet and Paneth cells, play a crucial role in strengthening the barrier function. They achieve this by secreting cytokines and antimicrobial peptides such as defensins that control the overgrowth of commensal and pathogenic bacteria.^43,46^

The goblet cells have recently been shown to have a novel gate-keeping role for the presentation of oral antigens to the immune system. Goblet cells deliver small intestinal luminal material to the lamina propria dendritic cells of the tolerogenic CD103^+^-type.^27^

Another type of specialized IECs is concentrated in the follicle-associated epithelium which overlays the luminal surface of lymphoid structures. These microfold cells or M cells are specialized epithelial cells that participate in inflammatory responses by capturing antigens in the luminal surface of the intestinal mucosa and transporting them to PPs through transcytosis for antigen presentation.^47^ These cells mainly recognize bacteria-derived molecules, through TLRs and NOD-like receptors (NLRs), which activate defense mechanisms through the secretion of cytokines and chemokines that signal to the underlying immune cells and induction of IgA-secreting plasma.^43^

Intestinal epithelial cells maintain a strong and intricate interaction with the intestinal immune cells. Interactions between IECs and macrophages and other immune cell types promotes a harmonious crosstalk mechanism that sustains a healthy environment.^43^

**Figure 6.**
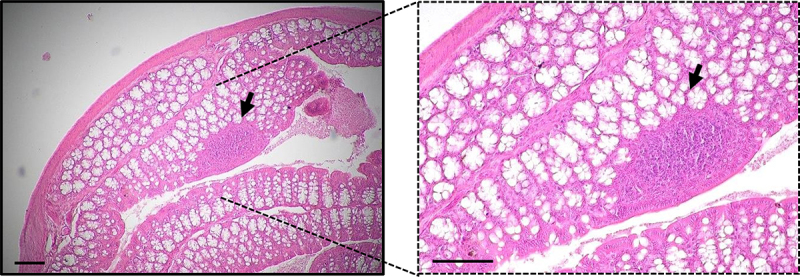
Representative photomicrograph of hematoxylin and eosin (H&E) staining in distal colon. Staining was performed in 5 μm sections from paraffin embedded colon from a C57Bl/6J mouse 20 weeks age fed with chow diet. GALT structure is marked with an arrow. Scale = 100 μm.

A network of intestinal innate and adaptive immune agents (dendritic cells (DCs), macrophages and lymphocytes, among others) reside within the epithelium and gut lamina propria. Moreover, lymphoid cells that include DCs, T cells, Treg cells and B cells can accumulate into gut-associated lymphoid tissue (GALT) that comprises the Peyer Patches (PPs) and Isolated Lymphoid Follicles (ILFs) depending on its location and composition.^43,46,48,49^ PPs are structured and highly organized lymphoid structures that are mainly located in the small intestine, whereas ILFs are more diffuse structures found in the small and large intestine.^46^ Photomicrograph of GALT structures in colon is represented in Figure 6. These sites serve as hubs for promoting tolerance to food antigens, regulating the balance of intestinal microflora, and warding off potential pathogens. Consequently, they serve as pivotal sites for triggering the adaptive immune response, capable of generating SIgA, which functions as a primary defense mechanism in the intestinal tissue.^50,51^

SIgA is mainly secreted by plasma cells located within mucosal membranes lining the gastrointestinal tract. It is produced in response to microbial- and food-derived antigens and plays different roles in intestinal mucosal secretions. It acts as first line of defense against pathogens and facilitates mucus surface colonization by commensal microbiota and regulates immune homeostasis.^52^

**Figure 7.**
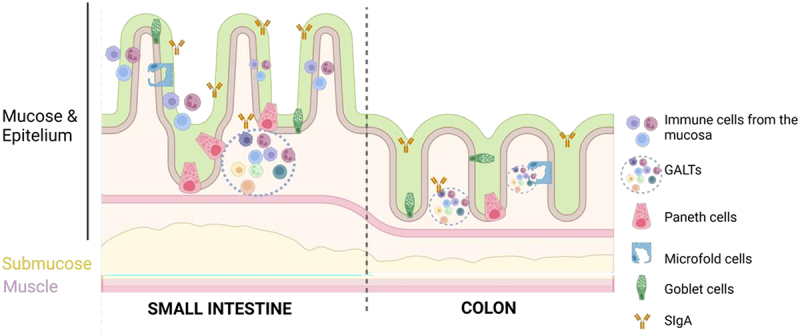
Immune barrier. Immune cells are mainly located in the gut mucose where secretory immunoglobulin a (SIgA) can be found. Gut barrier include macrophages, lymphocytes, paneth cells (more abundant in the small intestine) and microfold cells (M). Moreover, the gut-associated lymphoid tissue (GALT) is located in the lamina propria and can be classified into Peyer’s patches (PP) or isolated lymphoid follicles. Figure created with BioRender.com.

This combination of physical and biochemical defenses acts as a barrier against both commensal and pathogenic microorganisms (Figure 7).

### The microbial barrier

The microbial gut barrier is the third component of the gut barrier. The intestinal microbiota consists of highly diverse communities of prokaryotic and eukaryotic microorganisms as well as viruses. After birth the gut becomes colonized with comparably few different microorganisms and the ecosystem is relatively unstable. Thereafter, diversity of the microbiota increases and eventually results in the formation of a complex microbial ecosystem.^53^

Human adult microbiota is composed of 100 trillion microorganisms that include commensal bacteria, pro- and anti-inflammatory, pathogenic, and nonpathogenic bacteria, fungi, and viruses that maintain gut homeostasis.^54,55^ The intestinal microbiota increases in density from the small intestine to the colon, where it reaches approximately 10 trillion cells per gram of colonic content.^53^

Gut microbiome is a complex and dynamic community that keeps a symbiotic relationship with the host.^56–58^ During the life of an individual the microbiome changes in percentage and population. Under healthy and physiological conditions, it maintains host immune homeostasis.^56–58^ Some phyla are usually present in the gut microbiome: *Firmicutes, Bacteroidetes, Proteobacteria, Verrucomicrobia, Actinobacteria* and *Fusobacteria*.^59^

It provides efficient protection from infection by enteric pathogens (“colonization resistance”), stimulates maturation of the immune system and facilitates the conversion of non-digestible complex carbohydrates.^60,61^

Most often the microbiome takes part in metabolic processes including the fermentation of polysaccharides and the regulation of bile acid (BA) production. Besides, the contribute to the choline metabolism and the process of energy harvest, providing protection against pathogens or even stimulating the endogenous ethanol production.^62–64^

However, changes in the diet, drug toxicity, a proinflammatory environment and the presence of other microbiota members have been defined as the main microbiome modifiers and dysbiosis inductors.^65^

Hence, the excessive proliferation of some bacterial species, or the loss of some commensal bacteria, as well as variations in the total number of bacteria, is named dysbiosis, that is frequently associated to the pathogenesis of several inflammatory diseases and potential infections.^66,67^

## Gut barriers and microbiome crosstalk

The interactions among microbiome and mechanical and immunological components that constitute the gut barrier are essential for intestinal and systemic homeostasis.

### Microbiota and mechanical gut barrier interaction

Mucus metabolism is influenced by several factors, being one of them the microbiota that can affect the structure and function of the outer mucus layer. It has been described that germ-free animals have thinner mucus layer and fewer goblet cells.^68^ Meanwhile, some bacterial subproducts such as lipopolysaccharide (LPS) a cell wall component from gram-negative bacteria and peptidoglycan, cell wall component from gram-positive bacteria can stimulate mucus secretion. Conversely, some resident bacteria like *Akkermansia municiphila*, can break down the mucus to obtain energy for themselves and other commensal bacteria in a balanced stage.^69,70^ This process is more active when the diet is low in fiber, as fiber serves as energy source for the microbiota.^71^ Additionally, immune cells play a role in regulating mucus metabolism through the secretion of cytokines.^26^

One of the primary roles of gut microbiota is to obtain nutrients for the intestinal cells and break down undigested dietary products, such as fiber and protein.^61^ Specifically, by anaerobically fermenting complex carbohydrates that have not been digested, the gut microbiota can create short-chain fatty acids (SCFAs) like butyric, propionic and acetic acid. These SCFAs serve as an energy source for IECs, they can influence on cell proliferation and reduce cytokine production by neutrophils and macrophages, leading to an immunotolerogenic phenotype.^72^ Moreover, SCFAs can enhance the production of the mucus layer by modifying the transcription of mucin genes in goblet cells and can also encourage the reassembly of TJs, thereby strengthening the intestinal epithelial barrier.^73,74^

### Microbiota and immune system interaction

The gut microbiota also contributes to the development of the host’s immune system by producing metabolites, microorganism-associated molecular patterns (MAMPs), including pathogen-associated molecular patterns (PAMPs), and antigens.^75^ Bacterial translocation refers to the process by which pathogens or their products move from the intestinal lumen to mesenteric lymph nodes (MNL).^76^ LPS, which is present in gram-negative bacteria cell wall, is one example of a MAMP recognized by receptors on cells from the innate immune system, including TLRs. These receptors are pattern recognition receptors (PRRs) that are typically found on the surface of immune and intestinal cells and transmembrane proteins, and are capable of identifying bacterial, viral, or parasitic ligands.^77^ When activated by pathogens or commensal bacteria, TLRs can trigger an immune response through the induction of the nuclear factor- κB (NF-κB), a group of transcription factors involved in the production of inflammatory cytokines. This symbiotic interaction between commensal bacteria and the host immune system plays a protective role in maintaining intestinal homeostasis.^78,79^

## Gut and liver crosstalk

The concept of “gut-liver axis” had initially been proposed to describe the presence of antibodies directed against intestinal microorganisms and food antigens in the circulation of patients with liver cirrhosis,^80^ the common end stage of chronic liver disease (CLD).

Both gut and liver diseases are frequently associated with a perturbed regulation of gut-liver communication and dysbiosis. This fact, together with the disruption of mechanical gut barrier, altered immune homeostasis and imbalanced bile salts pool, triggers enhanced gut permeability “leaky gut” and systemic inflammation. Alterations of the structure and functions of the gut microbiota have major effects on the gut and liver. In fact, studies show that the microbiota has an ethiopathogenic role in gut and liver diseases and that, in turn, gut and liver disease alter the enteric microbiota composition.^81,82^

Yet, the clinical relevance of the gut-liver axis is not limited to aspects concerning microbiota and bacterial dissemination. Gut- and liver-derived mediators including cytokines, hormones, bile salts and other factors, directly link the function of both organs.

Moreover, liver disease disrupts gut homeostasis and leads to changes in gut microbiota composition and intestinal permeability, which correlates with the severity of liver dysfunction. Portal hypertension causes alterations in intestinal barrier function, allowing normally restricted substances to enter the bloodstream. The translocation of bacterial products or fragments triggers the immune system activation and inflammation. This process not only exacerbates liver dysfunction, but also initiates a series of reactions throughout the body, leading to a systemic inflammatory condition characteristic of advanced liver cirrhosis.^83^

### Metabolic crosstalk between the gut and the liver

**Figure 8.**
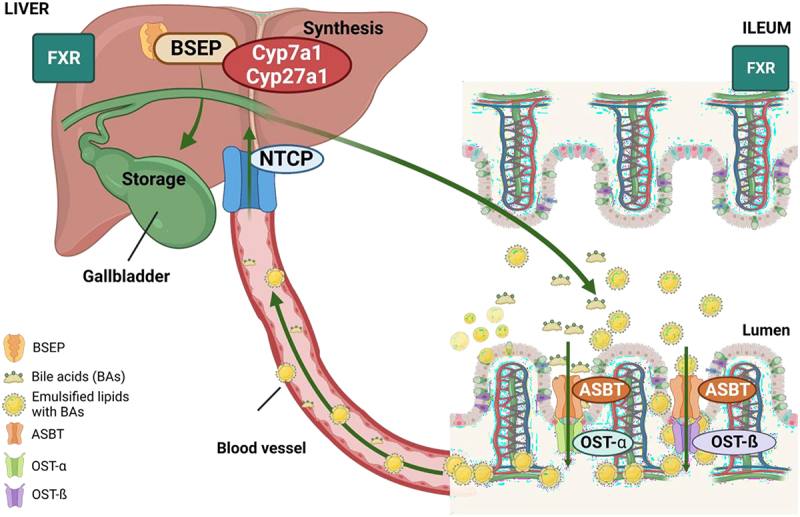
Enterohepatic BA cycle. Bile synthesis is performed in the liver by Cyp7a1 and Cyp27a11 enzymes. When synthesis is completed, BA are released in the bile canaliculi through the BSEP pump and stored in the gallbladder. In the ileum, bile salts are absorbed by ASBT and efluxed by OST-α/β to the circulation. Back to the liver they are uptaken by NTCP, a transporter located in the basolateral membrane of the hepatocytes. ABST, apical sodium-bile acid transporter; BSEP, bile salt export pump; FXR, farnesoid X receptor; NTCP, sodium taurocholate co-transporting polypeptide; OST, organic solute transporter.

BA are amphipathic molecules that are synthetized in the hepatocytes as primary BA, cholic acid (CA) and chenodeoxycholic acid (CDCA), that are transported from the canalicular side of the hepatocyte by the bile salt export pump (BSEP) along with phospholipids and cholesterol. Conjugated BA are actively transported into the ileum. After reabsorption, BA induce the synthesis of fibroblast growth factor 15/19 (FGF-15/19). They exit the basolateral side of ileocyte via the heterodimeric organic solute transporter (Ost-a/b). BA and FGF-15/19 are transported back to the liver via the portal blood. Conjugated BA are actively transported into the hepatocyte primarily by the Na^+^/taurocholate co-transporting polypeptide (NTCP). FGF-15/19 binds to and activates hepatic fibroblast growth factor receptor 4 (FGFR4), which in turn activates JNK signaling pathway. Activation of JNK downregulates the gene encoding cholesterol 7α-hydroxylase (CYP7A1), inhibiting BA synthesis^84^ (Figure 8).

During bile salt enterohepatic circulation, several hundred milligrams of BA escape this cycle and enter in the colon where they are metabolized by gut microbiota, generating secondary BAs. These substances and derived metabolites are passively absorbed from the colon and returned to the liver via the portal vein, where they will be re-conjugated to either glycine or taurine and join primary bile salt cycle.^84^

Briefly, one of the functions of BA is to emulate fats and bring them near the intestinal brush border membrane which results in fat absorption in the gut. The ratio of taurine to glycine BA depends on the diet in human, but not in rodents.^84^ In diet- and obesity-induced MASLD, high fat diet (HFD) can modify BA composition due to liver damage, which potentially has an impact on the gut microbiome.^85–88^

Moreover, BA have other metabolic actions and have been recognized as signaling molecules in the body through Farnesoid X Receptor (FXR) and TGR5, playing a key role in the control of hepatic de novo lipogenesis, very low density lipoprotein (VLDL) and plasma TG turnover. FXR is strongly expressed in the liver and the intestine, where is a regulator of BA enterohepatic circulation but can interact with fat metabolism. Some studies demonstrate that gut microbiota can also modify BA secretion though FXR, fostering lipid peroxidation and hepatic steatosis.^17,89,90^

It has been recently reported that FXR deficient mice are protected against diet-induced obesity. FXR suppression is alleviated by microbial metabolism of TβMCA and βMCA. Higher levels of TβMCA are related to lower levels of FXR activity.^91,92^ It has been described that conventionally raised microbiome mice under a HFD feeding increase lipid accumulation in the liver due to higher expression in *Cd36, ApoC2* and *Vldlr* genes when compared to germ-free (GF) mice.^91^ These data would suggest that, in part, gut microbiota can interact to FXR and partially induce hepatic steatosis.

It has been also described that FXR activation inhibits *Srebp1-c* expression, and increases insulin sensitivity, reducing obesity and supressing inflammation. Moreover, the activated TGR-5 would bind to secondary BAs, stimulating GLP-1 and playing an important role in feeding signals and glucose homeostasis.^93^

### Immunological crosstalk between the gut and the liver

Gut microbiome is a particularly important mediator of the gut-liver axis.^94^ Microbiome serves as the primary mechanism of interaction with the liver via TLRs. Rodents express 13 TLRs, whereas humans do have only 10. The presence of multiple widely expressed TLRs allows for the recognition of various microorganisms, triggering the appropriate immune response by the innate immune system.

PAMPs consist of microbial molecular structures like LPS from Gram-negative bacteria, lipoteichoic acid and peptidoglycan (PGN) from Gram-positive bacteria, lipoglycans, lipopeptides, and lipomannans from mycobacteria, zymosan from yeast, as well as DNA from viruses and bacteria. Damage-associated molecular patterns (DAMPs) include components of the extracellular matrix and plasma membrane, nuclear and cytosolic proteins, and elements from damaged organelles.^95^

**Figure 9.**
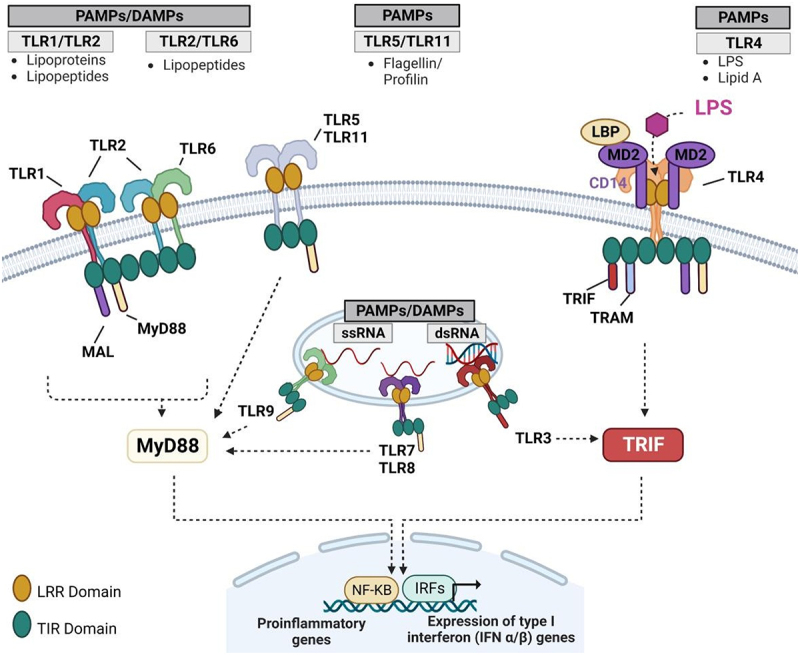
TLR types. TLR1, 2, 4, 5 and 6 bind to molecules associated with bacterial membranes. Concretely, TLR4, one of the most studied TLRs, binds to the LPS from the Gram-negative bacteria cell wall. TLR3, 7, 8 and 9 recognize viral, bacterial, or endogenous nucleic acids. Immune response initiation is mediated by TLR activation. DAMP, damage associated molecular patterns; LPS, lipopolysaccharide; PAMP, pathogen associated molecular patterns; TLR, toll-like receptor.

Each TLR possesses the ability to recognize specific molecular patterns. TLR1, TLR2, TLR4, TLR5, and TLR6 bind to molecules associated with bacterial membranes such as LPS, lipoprotein, and PGN. On the other hand, TLR3, TLR7, TLR8, and TLR9 detect viral, bacterial, or endogenous nucleic acids. TLR4, in conjunction with TLR2, can identify antigens from bacteria, fungi, parasites, viruses, and DAMPs. LPS, which is a constituent of the cell wall in gram-negative bacteria and interacts with TLR4, is one of the extensively studied mediators of host-microbe interactions^95^ (Figure 9).

## MASLD and Gut-barrier disruption

MASLD development is intricate and involves multiple factors. A more comprehensive “multiple hit model” has been recently proposed to explain disease’s onset and has gained acceptance. According to this model, the initial hit leads to increased liver fat levels, followed by the influence of various factors such as IR, gut microbiota, and genetic/environmental elements. These factors collectively affect the inflammatory environment within hepatocytes.^96^

Fat accumulation, hepatocyte injury, and particularly, intestinal barrier damage are crucial elements in the pathophysiology of MASLD. The intestinal barrier plays a vital role in absorbing essential nutrients and preventing the intrusion of microorganisms from the gut lumen. When alterations occur in the intestinal barrier function, it leads to increased intestinal permeability, which significantly contributes to the initiation and progression of intra and extrahepatic damage in MASLD.^96,97^ MASLD is linked to gut barrier disruption, changes in TJs, rise in intestinal permeability and dysbiosis.

### Mechanical barrier disruption in MASLD

Loss of mucus and chemical substances in the gut barrier can lead to bacterial overgrowth in the GI tract, disrupting gut homeostasis and permeability.^96^

Recent studies have demonstrated that heightened inflammation in the intestinal mucosa and damage to the intestinal epithelial barrier increase the possibility of microbial translocation contributing to MASLD. Concretely, disruption and alterations in the TJs has been described in Metabolic dysfunction Associated Steatohepatitis (MASH) patients and rodents’ model.^98,99^ Under conditions of hypoxia and inflammatory stimulation, TJ proteins exhibit contractions and shift to the cytoplasm. Consequently, the cell pores expand significantly, leading to increased permeability of the intestinal mucosa. This, in turn, allows for the translocation of intestinal bacteria and the release of bacterial byproducts (e.g., LPS) into the bloodstream and liver through the portal system. As a result, liver Kupfer cells (KCs) are stimulated leading to the release of inflammatory factors.^96^

The disruption of the GVB plays a crucial role in facilitating the entry of gut bacteria and bacterial products into the bloodstream.^42^ PV-1 expression, which serves as a marker of GVB permeability, is elevated during pathogenic events like the systemic dissemination of bacteria, including MASH among others. Studies have revealed that GVB disruption is noticeable during the early stages of MASH. Enteric pathogens have been found to breach the GVB by interfering with the WNT/β-catenin pathway in endothelial cells.^37,38^

### Immunological barrier disruption in MASLD

The primary constituents of the immune barrier are lymphocyte- and plasma cell-secreted IgA. IgA exhibits a specific affinity for Gram-negative bacteria present in the GI tract. However, when the intestinal mucosa is impaired, the functionality of IgA is hindered, which, in turn, facilitated bacterial translocation within the intestine and contributes to inflammation.^100,101^

The gut-liver axis plays a pivotal role in the development of MASLD. Sterile inflammation triggered by DAMPs and MAMPs is recognized a significant factor in causing liver damage.^102^

Recent research has revealed elevated levels of serum IgA in MASLD patients, which is produced by plasma cells in secondary lymphoid organs. Long-term inflammation and fibrosis in both human and mouse models of MASLD were linked to liver-resident IgA producing cells expressing PDL1, hindering body’s ability to effectively avoid cancer.^103,104^ In a mouse model of MASH, B cells within the liver stimulated by microbial factors from the hut, contributed to liver inflammation and fibrosis.^105^

Moreover, individuals with MASLD exhibit a decrease in FOXP3-expressing regulatory T cells (Tregs), alongside an increase in Th1 and CD8+T cells within the lamina propria of the gut.^106^ Mast cells (MCs) which are immune cells located in the intestinal barrier, play a role in regulating both innate and adaptive immunity. These MCs release cytokines, histamine and proteases, which can affect the integrity of the intestinal barrier. The primary proteases, trypsin and chymases, are responsible for ZO-1 cleavage, reducing the expression of JAM-A, and increasing the permeability of the gut epithelium.^107^ Recent research suggests that histamine can elevate the permeability of the intestinal epithelium and the translocation of gut bacteria in murine models.^108^

SIgA produced by lymphocytes and plasma cells plays a crucial role in immune function at the gut barrier. There is evidence indicating that serum IgA levels are notably higher in individuals with severe NASH compared to those in the early stages of the disease, and this elevation is linked to advanced fibrosis.^103,109^

### Microbial barrier disruption in MASLD

High- fat, cholesterol, and refined carbohydrate diet, currently known as western diet (WD) can induce microbiome changes and gut dysbiosis, decreasing populations of commensal bacteria that keep gut barrier integrity and increasing other bacterial populations, such as gram-negative bacteria known to have a proinflammatory effect and to induce a harmful environment.^8,110,111^ Hence, these facts could induce an increase in gut permeability, triggering the activation of TLRs family and its consequent inflammation by LPS and endotoxin among other bacterial subproducts.^54,65^

Consistently, some studies have identified that obesity, high-kcal, fat, and carbohydrate diet can lead to gut dysbiosis, gut damage and metabolic disarrangements.^112,113^ Others have described that obese an overweight adults have less microbial gene count.^114–116^ Moreover, lower *Firmicutes/Bacteroidetes* ratio has been correlated with lean humans when they were compared to obese individuals.^116,117^

Clinical data from the past two decades has convinced scientists that MASLD patients exhibit a reduced gut microbiome signature, as indicated by increased percentage of *Proteobacteria*, *Enterobacteria*, *Escherichia*, and *Bacteroides* species and decreased the percentage of *Firmicutes* species in the gut microbiome profile.^118^

As a result, gut permeability could be directly affected by high fat contents but also by dysbiosis. Nonetheless increased gut inflammation can also induce by itself gut damage, dysbiosis and an increase in gut permeability. Despite dysbiosis has been pointed out as one of the primary mechanisms by which the altered microbiome induces gut inflammation, and consequently alter the gut permeability, it remains unclear the beginning of this feedback loop.

## Diet, metabolism and microbiome, feedback loop in the development of obesity and MASLD

Lately, the role of SCFAs have been described as a key in the development of obesity and gut dysbiosis. Overproduction of SCFAs can be stimulated by WD consumption and could increase the energy intake (kcal) coming from the food.

SCFAs origin is the fermentation of some carbohydrates that have not been digested. Propionate, acetate, and butyrate are the most common SCFAs. While butyrate constitutes an energy source for colon epithelial cells and exhibits immunomodulatory and anti-inflammatory properties that contribute to the homeostasis of the gut barrier, acetate has been described as an obesogenic SCFA. Overall, colonic derived SCFAs account for 10% of harvested energy from the diet, with acetate being the main source of energy. Hence, the metabolic capacity of the microbiome can increase the energy extraction from the diet through the fermentation of complex polysaccharides present in the WD to SCFAs.^119,120^

Other studies show that obese profiles might also exhibit low levels of SCFAs. In this situation high content of hydrogen sulfide (H_2_S) has been detected. H_2_S can be produced either by the host or the microbiome itself. High level of this component can be explained by the direct impact of specific fat sources in BA production. Despite of its effect in metabolism it is not clear, high saturated fats diets have been related to increased levels of taurine-conjugated bile salts and obesity.^121^

Several recent studies have demonstrated that the specific combination of metabolic cofactors composed of L-carnitine (an enhancer of FFA uptake across the mitochondrial membrane), nicotinamide riboside (NAD+ precursor), n-acetyl cysteine, and betaine (glutathione precursors and betaine a methyl donor) is a promising treatment against MASLD.^122^ Such multi-ingredient supplementation improves pathological MASLD features in the liver, reducing inflammation, steatosis, and IR.^123,124^ Moreover, multi-ingredient supplementation administrated to diet-induced MASLD mice is able to ameliorate gut morphological changes, increase epithelial cell proliferation and the number of goblet cells, restore TJ barrier integrity, and reduce intestinal inflammation by improving intestinal microbiota composition diversity, as well as by modulating short-chain fatty acids (SCFAs) concentrations in feces. In addition, supplementation with metabolic cofactors induces the reduction of gut microbiota-derived propionate levels linked to decreased levels of Firmicutes contributing to the prevention of propionate-induced lipid accumulation in the liver.^8^

The amino acid histidine is another key energy source for the microbiota, scavenging it from the host. Different metabolic medical conditions, such as obesity, heart failure, and hepatic steatosis are connected with decreased histidine levels.^125,126^ Importantly, plasma histidine levels are negatively associated with several bacterial families that are also increased in MASLD, in particular from the phylum Proteobacteria. Patients with a higher degree of liver steatosis have higher clr-transformed levels of *hutH*, *hutU*, and *hutI*, suggesting a higher catabolism of histidine by the gut microbiota, associated with lower histidine plasma levels. Notably, histidine supplementation improved MASLD in different animal models (diet-induced MASLD in mouse and flies, *ob*/*ob* mouse, and ovariectomized rats) and reduced *de novo* lipogenesis.^127^

Not only subproducts of microbes but also the gut microbes by themselves have an impact in host metabolism. It is known that certain profile of gut microbiota could raise the activity of lipoprotein lipase (LPL), a fat uptake enzyme. Moreover, AMP-activated protein kinase (AMPK), an energy metabolite in the liver and skeletal muscle, can be also disrupted by gut microbiota, and its decrease would reduce the lipid oxidation.^120^ Therefore, the gut microbiota environment together with a specific diet can have an impact on host metabolism.

As previously mentioned, one of the inflammatory pathways in MASLD is mediated by KCs that can be activated by TLRs. Host inflammation can be increased by gut microbes. Previous work shows that the loss of specific TLRs or use if innate immune adaptors as MyD88 exhibit a protection against HFD-induced obesity. Moreover, it has been revealed that TLR4 activation is sensitive to saturated fatty acid metabolism and hepatic and serum lipid profile induced by HFD.^128^

Moreover, it has been previously described that ethanol endogenous production boosted by dysbiosis directly contributes to MASLD. Microbiota fermentation of undigestible carbohydrates from the diet can induce alcohol endogenous production in the intestinal lumen. Endogenous ethyl alcohol reaches the liver by the portal vein which contributes to induce liver damage that aggravates MASLD pathology. Liver metabolizes the ethanol, product of fermentation by ADH and cytochrome P450 isozymes, contributing to mitochondrial dysfunction and being considered a causative factor for the development of MASLD.^129–131^

## Clinical management of MASLD

Despite extensive research on understanding the pathophysiology of MASLD, no targeted therapies are yet available.^132^

### Dietary intervention for MASLD management

Epidemiological research has demonstrated the link between eating habits and liver disease. It is now widely acknowledged that diet plays a crucial role in the development of CLD and is also a fundamental aspect of its management. Recent investigations have suggested that a diet high in sugar, saturated fats, and cholesterol contributes to the progression and emergence of MASLD. Conversely a diet abundant in fruits, protein, polyunsaturated fats and vegetables is associated with a reduced risk of MASLD.^133^ Some studies proposing dietary interventions advocate a regimen in which carbohydrates primarily come from cereals, fruits, and vegetables, protein accounts for approximately 12% of the total daily energy intake, and fat is minimized and derived from vegetables. Besides, these interventions emphasize the avoidance of alcohol and smoking. Changing dietary habits highlight the significant role of diet in the treatment of liver disease.^133,134^

The interplay between the gut microbiota and dietary habits is a dynamic process where the gut microbiota influences the host’s response to diet, and, in turn, the host can impact the gut microbiota through change in dietary patterns.^135^

Diet plays a role in shaping the composition, diversity, and richness of the gut microbiota over time. Human studies have demonstrated noticeable shifts in the gut microbiota just 24 h after transitioning from a high-fat/low fiber to a low-fat, high-fiber diet.^136^ Balanced and *healthy* diet enhances the integrity of the gut barrier, increases mucus production, lowers luminal pH, and reduces the leakage of microbes into the bloodstream. This leads to improved insulin sensitivity and increase in anti-inflammatory markers.^113,137^

Dietary fiber plays a crucial role in creating an optimal gut environment that supports the flourishing of beneficial bacteria (eubiosis), resulting in beneficial physiological effects such as reduced plasma cholesterol and glucose levels. Moreover, it enhances the presence of SCFAs- producing species as *Akkermansia, Bifidobacterium, Lactobacillus, Ruminococcus, etc*..^138^ Furthermore, these substances serve as an energy source for enterocytes, increase mucus production, contribute to maintain immune homeostasis, and act as important signaling molecules systemically.^139^ Clinical trials of dietary interventions mentioned above have been summarized in Suppl. Table. S1.

### Physical exercise for MASLD management

Previous discussions have highlighted the association between CLD and its worsening in the presence of obesity and MS features. Moreover, individuals diagnosed with both conditions are at heightened risk of developing cardiovascular diseases, such as myocardial infarction and stroke. Due to the increased susceptibility of these patients to severe liver damage and cardiovascular issues, it is of utmost importance to prioritize weight loss and lifestyle changes in their overall care.^140^

Several studies indicate that physical exercise can directly benefit the liver and indirectly affect it through non-hepatic pathways.^141–143^ Advantages of physical activity, including structured exercises extend beyond and serves as a fundamental treatment for patients with MASLD. Both aerobic and resistance training have been shown to effectively reduce hepatic steatosis and alleviate the cardiovascular risk associated with MASLD.^142,143^

Recent studies demonstrated that athletes had a higher diversity of gut microorganisms; however, the mechanisms remain unclear.^144^ In line with these data, cardiorespiratory fitness has been reported to improve gut barrier integrity in patients undergoing coronary artery disease.^145^ Some animal studies in rats and mice demonstrated that exercise effectively counteracted HFD-induced microbial imbalance, leading to intestinal barrier preservation, which in turn prevented deregulation of gut liver axis and improved BA homeostasis.^141,146,147^ Specifically, some reports indicated that both short- and long-term exercise programs, spanning a range of intensities, can enhance the body’s ability to counter oxidative stress, stimulate the turnover of lymphocytes, and boost the expression of anti-inflammatory cytokines within the intestinal lining.^148^ It is proposed that exercise promotes intestinal motility, potentially leading to the shedding of loosely attached microbes from the GI epithelium.

Physical exercise fosters the proliferation of other beneficial microorganisms that play a role in maintaining healthy mucosal immune system and gut barrier balance.^149^

Furthermore, animal research indicates that engaging in aerobic exercise improved intestinal mucosal morphology and resulted in the upregulation of claudin 1 and occludin. This was accompanied by a decrease in endotoxemia, suggesting that exercise training has the potential to partially rehabilitate the function of the intestinal barrier.^147,150^ The studies and interventions citated above have been summarized in Suppl. Table. S2.

### Pharmacotherapy. FXR modulators

The lipotoxicity, the inflammation and the fibrosis are the most described mechanisms that contribute to MASH development. One of the target pathways is the regulation of BA synthesis, specifically the modulation of FXR. As it has mentioned above, FXR us a central molecule in the BA metabolism, and it manages post-prandial stage signals, limits the lipogenesis and the gluconeogenesis.^17,89^

Several studies with FXR agonists have been published. In 2021 Clifford et al. demonstrated in murine that the use of *GSK2324* a FXR agonist reduced lipid uptake as well as decreased lipogenesis, as a result hepatic steatosis was significantly reduced.^151^ Moreover, other FXR agonists have been used in randomized control trials as cicloflexor (GS-9674) in patients with MASH, decreasing hepatic steatosis, the transaminases in serum and the circulating BAs. However, patients suffered from pruritus as side effect.^152^ The use of MET-409, a structurally novel molecule that agonists FXR has also reported beneficial effects in lipid accumulation in the liver and lower levels of hepatic transaminases, however, the use of high doses reports high expression of FGF-19, a potential indicator of drug accumulation that could also explain the increase of LDL-c cholesterol and pruritus. However, the use of lower doses of MET-409 could balance its adverse or beneficial effects.^153^

Nevertheless, it is not clear whether FXR should be agonized or antagonized. Several studies demonstrated that FXR antagonists can be used also as a potential drug for MASLD. Concomitantly, TGR5 agonists, antagonizing FXR can stimulate adipose tissue thermogenesis, boost energy metabolism, and reduce inflammation.^154^ Moreover, they have been reported to alleviate obesity and MS in mouse models.^155^

All in all, FXR agonists can be effective in the reduction of the accused MASH-associated lipotoxicity, however more translational studies are needed due to the differences between animal models and humans, side effects should be reduced, and more translational markers included. Furthermore, it should be elucidated whether its agonism results more interesting than its antagonism. Studies and clinical interventions mentioned above have been summarized in Suppl. Table. S3.

### Microbiome modulation in MASLD

Close connection between the gut and the liver (gut-liver axis) and dysbiosis as one of highlighted features in CLD, sheds light on another putative therapy for the management of the disease: the restauration of intestinal microbial diversity.

Preclinical and clinical studies suggest that the microbiome could be used as a novel target to alleviate the pathophysiology of MASLD. One of the most common effects that microbiota changes have on the host, is the release of bacterial metabolites that play important roles in energy homeostasis. After food ingest the body releases compounds named BA for digestion. BA are known to control bacterial overgrowth and maintain intestinal barrier function.^156^ Moreover, bacteria also influence in BA metabolism transforming the primary BA into secondary BA in the colon, modulating FXR and TGR5 expression, having a direct effect on glucose tolerance and homeostasis, insulin sensitivity, lipid metabolism, triglycerides (TG) and cholesterol levels and energy expenditure by the host.^84^

#### Prebiotics, probiotics and symbiotics

Probiotics are a group of beneficial microorganisms that actively colonize the human gut and reproductive system, aiming to improve the imbalanced microbiota of the host. Studies have shown that supplementing with probiotics can decrease the presence of pathogenic bacteria by absorbing endotoxins, enhance the balance of microecology, and reduce the production and entry of harmful substances into the liver. As a result, probiotics play a preventive and alleviating role in the pathological process of MASLD.^157^

On the other hand, prebiotics are indigestible food ingredients that can effectively improve host health by selectively stimulating the growth and activity of specific bacterial colonies. They work by influencing the activity of probiotics and have a positive impact on the human body.^158^ When probiotics and prebiotics are combined, the are named symbiotics and their collective effects are mutually beneficial.^158^

Preclinical and clinical studies suggest that the use of probiotics could alleviate the pathophysiology of MASLD. Some animal studies use combination of *Bifidobacterium* and *Lactobacillus*. The cholesterol and TG content in the liver was significantly reduced together with the hepatic transaminases in serum.^159^

Other studies suggest the use of probiotics in combination with metronidazole, boosting the effect on lipid profile, liver function, oxidative stress, and inflammatory markers in rats with MASLD.^160^

The administration of *Lactobacillus* and *Bifidobacterium* as a probiotic in pilot patients despite no significant effect was demonstrated on MASLD, the mucosal immune function was stabilized.^161^ Other clinical trials reported minor differences with the use of probiotics to treat obesity a MASLD. Despite the single use of probiotics to treat MASLD pathophysiology reports no significant differences, it is suggested its combined use with other treatments.^7^

Furthermore, the beneficial effect may be a result of a combination of actions, which may be related to the enzymes or metabolites produced by specific strains.^162^ Regarding intestinal barrier function, increasing evidence shows that probiotics stimulate immune function against enteric pathogens and at the same time regulates intestinal inflammation by PPARγ expression.^162,163^ Besides, probiotics contribute to the improvement of intestinal barrier function by not only suppressing the host’s inflammatory response but also by altering the characteristics and secretion of intestinal mucus. The composition of intestinal microorganisms, leading to changes in the nature of mucus and increased secretion.^162^ For instance, *Limosilactobacillus reuteri*, present in probiotic formulations has been shown to enhance the intestinal barrier by increasing mucus thickness in a mouse model of colitis.^164^ Additionally, probiotics promote the expression and localization of TJ proteins and genes related to mucin production.^162^ All the studies cited above have been summarized in Suppl. Table. S4.

#### Faecal Microbiota Transplant (FMT)

Gut microbiota is associated to metabolic diseases, including obesity, IR, and MASLD as demonstrated by correlative studies that transplant microbiota from obese humans or mice into mice, inducing greater symptoms of MASLD in the recipient animals.^165,166^

In this line, FMT has been proposed as a treatment for MASLD. Animal studies report that autologous or orthologous FMT from lean healthy donors potentiate loss of body weight and adiposity, and beneficial effects on MS.^167,168^

Regarding the application of FMT as a treatment, single or combined, for MAFLD, few animal studies show that the total FMT from healthy donors to HFD animals resulted into an improvement of gut permeability and decreased steatosis and inflammation in the liver of recipients.^169^

In the clinical practice, FMT is only approved to treat recurrent *Clostridium difficile* infection.^170,171^ However, some randomized clinical trials that propose FMT application to improve obesity, MS and MASLD have been developed.^172–174^ Currently, these studies have some limitations as the reduced sample size and the heterogeneity of donors and recipients. Despite significant changes in bacterial populations, it is hard to find remarkable improvements in MASLD pathophysiology when FMT is used as single treatment. Most of the clinical trials show a tendency of body weight and adiposity reduction in long term FMT application. Concerning the effects of FMT in liver, fat accumulation in the liver was decreased.^174^

Some studies report that metabolic changes of FMT include enhanced linoleic acid metabolism contributing to the improvement of gut barrier integrity. Recent results show that effectively repaired microbiome through FMT applied to *E.* *coli K88* infection, would increase *Lactobacillus* populations that exerts properties of maintenance of the intestinal integrity barrier upregulating mucosal MUC expression levels, butyrate production and ZO-1 expression^175^ as well as in other studies with affected gut barrier.^176^ All the studies mentioned above have been summarized in Suppl. Table. S5.

## Future perspectives

MASLD stands out as the most prevalent CLD, having reached epidemic levels globally among both adults and children.^2^ Despite its widespread impact, there is currently no approved pharmacological treatment for MASLD.^132^

MASLD management is a complex condition associated with factors like obesity, arterial hypertension, IR, and abnormal lipid profiles, that typically requires an intricate intervention once diagnosed.^177^

Predicting the onset and progression of MASLD is still challenging, yet genetic assessment emerges as a promising avenue in the pursuit of precision and individual medicine. Advances in understanding the human genome, coupled with next-generation sequencing technologies, pave the way for fully integrated genomic medicine. This approach would enable the classification of patients into high or low-risk categories for disease onset or severity, facilitating therapeutic interventions.^177^

Recent studies redefine MASLD as a systemic disease with impacts extending beyond the liver. Inflammation and disturbances in hepatic metabolism are linked to alterations in the gut, activation of the gut-liver axis, and dysbiosis.^5^

The concept of gut liver axis constitutes other unexplored via of treatment, suggesting that a healthier gut would enhance the hepatic function and vice versa. Some innovative treatments in research already incorporate this notion, particularly the microbiome modulation therapies. Strategies such as probiotics or FMT have being considered, viewing bugs as potential therapeutic agents. However, FMT for this purpose remains clinically unapproved, necessitating further studies to standardize procedures and ascertain its actual beneficial effects on the disease, alone or in combination with other treatments or recommendations.^178^ Additional approaches involve the use of drugs targeting the microbiome, such as specific antibiotics of phages. These aim to promote the growth of bacteria with favorable metabolic effects for the host, and to avoid the presence of harmful bacteria.^179,180^ These particular strategies also seek to provide individualized treatments, considering the significant variations in the microbiome among individuals.

Importantly, another aspect of consideration is the recent shift in the definition of fatty liver disease, incorporating the term MAFLD alongside NAFLD, having implications for diagnosis and prevalence. MASLD prevalence surpasses that of NAFLD, carrying a higher risk of overall mortality and increasing the variable factors among patients.^181^ Thus, diagnosis challenges go beyond the requirement to differentiate pure MASLD from alcoholic liver disease (ALD), as clinicians frequently observe a combination, referred as Metabolic associated liver disease in combination with increased alcohol intake (MetALD), that constitutes a big gray area in the hepatology field.^4,182,183^

## Conclusions

The development of MASLD is a complex process influenced by various factors, not limited to the liver. The integrity of the intestinal barrier is crucial for nutrient absorption and defense against microorganism intrusion from the gut. Disruptions in the intestinal barrier result in increased permeability, contributing significantly to both intrahepatic and extrahepatic damage in MASLD. This condition is associated with gut barrier dysfunction, changes in tight junctions, elevated intestinal permeability, and dysbiosis. Impairment of the intestinal mucosa is linked to compromised immune barriers, leading to heightened inflammation and intestinal damage, ultimately increasing gut permeability.

The interplay between the immune and mechanical gut barriers is closely intertwined with the gut microbiota. The intricate relationship between the adult human microbiota and the host is essential for maintaining gut homeostasis and immune function. A healthy microbiota consists of a balanced array of beneficial and harmful microorganisms that interact dynamically with their environment. The gut microbiota contributes not only to the maturation of the immune system and protection against pathogens but also influences the mechanical and immunological aspects of the gut barrier. It affects mucus composition, generates essential metabolites, and participates in antigen recognition and immune modulation, all of which are vital for barrier function. Dysbiosis, the disruption of this delicate equilibrium, has emerged as a significant factor in the pathogenesis of various inflammatory disorders and potential infections.

Current clinical management of MASLD primarily relies on dietary interventions and physical exercise. These strategies have demonstrated favorable effects on liver and gut physiology, as well as the restoration of a balanced microbiome. Recent research suggests the microbiome as a potential target that can complement existing clinical therapies to enhance their benefits. Nevertheless, the exact benefits of FMT and the intricate microbiota-host interactions remain unclear. In-depth research in this area is warranted to assess the potential of microbiota modulation in clinical pathways and to optimize existing procedures.

## Supplementary Material

Breaking the barriers SUPPL.docx

## Data Availability

The authors confirm that the data supporting the findings of this study are available within the article.

## List of Abbreviations

ALD: Alcohol-associated liver disease
AMPK: AMP-activated protein kinase
BA: Bile acid
BSEP: Bile salt export pump
CA: Cholic acid
CDCA: Chenodeoxycholic acid
CLD: Chronic liver disease
DAMP: Damage-associated molecular pattern
ECM: Extracellular matrix
FGF: Fibroblast growth factor
FGFR: Fibroblast growth factor receptor
FL: Fatty liver
FMT: Fecal microbiota transplant
FXR: Farnesoid X receptor
GALT: Gut associated lymphoid tissue
GF: Germ free
GI: Gastrointestinal
GVB: Gut vascular barrier
HCC: Hepatocellular carcinoma
H&E: Hematoxylin and eosin staining
HFD: High fat diet
HSC: Hepatic stellate cell
ICAM −1: Intercellular adhesion molecule-1
IEC: Intestinal epithelial cell
IgA: Immunoglobulin A\
ILF: Isolated lymphoid follicles
IR: Insulin resistance
KC: Kupfer cell
LPL: Lipoprotein lipase
LPS: Lipopolysaccharide
MAMP: Microorganism-associated molecular patterns
MASH: Metabolic dysfunction associated steatohepatitis
MASLD: Metabolic dysfunction associated steatotic liver disease
MetALD: Metabolic associated liver disease and increased alcohol intake
MLN: Mesenteric lymph nodes
MNL: Mesenteric lymph nodes lymphocytes
MS: Metabolic syndrome
MUC2: Mucin-2
NAFLD: Non-alcoholic fatty liver disease
NASH: Non-alcoholic steatohepatitis
NF-κB: Nuclear factor- κB
NLR: NOD-like receptors
NTCP: Na+/taurocholate cotransporting polypeptide
OST: Organic solute transporter
PAMP: Pathogen-associated molecular patterns
PGN: Peptidoglycan
PPs: Peyer Patches
PRR: Pattern recognition receptors
PV-1: Plasmalemma vesicle-associated protein-1
ROS: Reactive oxygen species
SCFA: Short chain fatty acid
sIgA: Secretory Immunoglobulin A
TG: Triglyceride
TJ: Tight junction
TLR: Toll-like receptor
VCAM: Vascular cell adhesion molecule
VLDL: Very low density lipoprotein
WAT: White adipose tissue
WD: Western diet
ZO: Zona occludens.
