# Supplementary material for: Breaking the barriers: the role of gut homeostasis in Metabolic-Associated Steatotic Liver Disease (MASLD)
Source: Gut Microbes. 2024 Mar 21;16(1):2331460. doi: 10.1080/19490976.2024.2331460 (PMC10962615; doi:10.1080/19490976.2024.2331460)
Supplement: Breaking the barriers SUPPL.docx [file KGMI_A_2331460_SM8967.docx]

**Breaking the barriers: The role of gut homeostasis in Metabolic-associated steatotic liver disease (MASLD)**

# SUPPLEMENTARY MATERIAL

**Table of contents**

1. [SUPPLEMENTARY TABLES 1](#_bookmark0)

[Suppl. Table 1. Dietary intervention studies in CLD 1](#_bookmark1)

[Suppl. Table 2. Physical exercise intervention studies in CLD. 2](#_bookmark2)

[Suppl. Table 3. Pharmacotherapy intervention studies in CLD. 3](#_bookmark3)

[**Suppl. Table 4.** Microbiome modulation intervention studies in CLD: Probiotic](#_bookmark4) [administration 5](#_bookmark4)

[Suppl. Table 5. Microbiome modulation intervention studies in CLD: FMT 6](#_bookmark5)

1. [SUPPLEMENTARY MATERIAL AND METHODS 9](#_bookmark6)

[Maintenance of mice and animal experimentation 9](#_bookmark7)

[Mice sacrifice, gut extraction and colon processing 9](#_bookmark8)

[Immunofluorescence staining 9](#_bookmark9)

[Hematoxylin and eosin staining (H&E) 10](#_bookmark10)

# SUPPLEMENTARY TABLES

## Suppl. Table 1. Dietary intervention studies in CLD.

| **Human studies** | | | |
| --- | --- | --- | --- |
| Population | Characteristics of the intervention | Results | Ref. |
| 61 patients diagnosed with CLD (39 men and  22 women), mostly between  51 and 65 years old | ESPEN dietary recommendations:  60-65%  carbohydrates, 12-  15% protein, fat reduces as much as possible. Food rich in vegetables and fruits. Salt in  moderation. Avoid | The prescribed diet regime has an effect on the treatment: 1. the proportion of respondents who have worsening disease was reduced, 2. a high proportion of respondents have improved or partially  improved compared to | Hajdarevic, B., et al. 129 |

|  | grilled or fried dishes, cooked or fried fat.  No smoking. No alcohol. | the beginning of the diet. |  |
| --- | --- | --- | --- |
| Cross-sectional analysis of 98 healthy volunteers | Different diet interventions in healthy subjects  followed by  16*S* rDNA stool analysis | Fecal communities  were strongly associated with long- term diets, particularly protein and animal fat (*Bacteroides*) versus carbohydrates (*Prevotella*).  Microbiome composition changed detectably within 24 hours of initiating a high-fat/low-fiber or low-fat/high-fiber diet. | Gary D. Wu., et al. 131 |

**Suppl. Table 2.** Physical exercise intervention studies in CLD.

| **Animal studies** | | | |
| --- | --- | --- | --- |
| Population | Characteristics of the intervention | Results | Ref. |
| 21-day-old Wistar rats | Animals were fed a HFD or control diet for  6 weeks and subsequently split in two groups: sedentary or exercise group (combined aerobic and resistance training). | Exercise attenuated MS, modulated the disrupted hepatic lipid metabolism, changed gut microbiota and improved the gut barrier. In addition, training protocol increased *Parabacteroi des*, *Bacteroides*  and *Flavobacterium* ge nera, correlating with a beneficial metabolomic profile,  whereas *Blautia*, *Dysg onomonas* and *Porphy romonas* showed an opposite pattern. | Carbajo- Pescador, S., et al. 142 |
| 6-week-old C57BL/6NTac  male mice. | Animals were fed a normal or HFD for 12 weeks and randomly assigned in two groups: sedentary and exercise for another 12 weeks. | Exercise ameliorated gut barrier integrity and modulated host microbiome. Specific phylotypes were observed in stool of exercise group,  including *Faecalibacter* | Campbell, SC., et al. 145 |

|  |  | *ium*  *prausnitzi*, *Clostridium*  spp.,  and *Allobaculum* spp |  |
| --- | --- | --- | --- |
| **Human studies** | | | |
| Population | Characteristics of the intervention | Results | Ref. |
| A meta-analysis of 10 randomized controlled trials. | study systematically reviewed self- controlled case series from randomized controlled trials  evaluating the therapeutic effect of transition from a sedentary lifestyle to  regular physical exercise | Altering a sedentary lifestyle to a lifestyle with regular exercise can slightly improve the levels of liver enzymes, blood lipids, blood glucose, insulin resistance, and body mass index in MASLD patients. | Ma, Q., et al.  138 |
| 41 individuals  (70% men, 30%  women), age =  62.7± 9.35 | Adults with coronary artery disease underwent a moderate-intensity  12-week cardiac rehabilitation exercise program. | Increase in  cardiorespiratory fitness during exercise program resulted in a relative improvement in a biomarker of gut barrier integrity (FABP2  in serum). | Feng, V., et al. 140 |

**Suppl. Table 3.** Pharmacotherapy intervention studies in CLD.

| **Animal studies** | | | |
| --- | --- | --- | --- |
| Population | Characteristics of the intervention | Results | Ref. |
| 8-week-old male mice of different backgrounds depending on the experiment: wildtype, *Fxr-/-, Shp-/-*, *Srebpc -/-, Pparα-/-, Fxrfl/fl.* | Animals were fed a control diet or WD for 8 weeks and then split in two groups where FXR agonist GSK2324 or placebo was administered. | Activation of FXR decreases hepatic triglycerides through two distinct  mechanisms. First, via bile-acid-dependent decreases in intestinal lipid absorption and second, through selective changes in lipogenesis. | Clifford, BL., et al. 146 |
| 5-6-week-old Lepob/ob mice. | Animals were fed with a chow diet or AMLN diet ((high fat with trans-fat, cholesterol and fructose) for 9 or 15 weeks and dosed  either with FXR/TGR5 | INT-767 improved steatohepatitis severity, ballooning degeneration, inflammatory infiltrates and fibrosis stage. In  the comparative study | Roth, JD., et al. 149 |

|  | receptor agonist (INT- 767) or obeticholic acid. | INT-767 exerted  greater therapeutic potency and efficacy than the obeticholic acid. |  |
| --- | --- | --- | --- |
| 6-week-old male mice of different strains: wild type C57BL76,  *Cyp27a1-/*-, *Tgr5-/-*  . | Animals were fed with HFD for 8 weeks before performing the studies. Next the obese mice were administered Ft1, a TGR5 antagonist, in a high dose 100mg/ 100g diet and 50mg/100g diet. | Ft1 is a TGR5 agonist but FXR antagonist to alleviate high fat diet- induced obesity and IR in mice. On one hand, Ft1 activates intestinal *Tgr5* to enhance intestinal GLP-1 release. On the other hand, Ft1 increases the hepatic BA production by suppressing  intestinal *Fxr*. | Ding, L., et al.  150 |
| **Human studies** | | | |
| Population | Characteristics of the intervention | Results | Ref. |
| Double-blinded placebo- controlled trial in  11 patients awaiting gallstone surgery. All  patients had symptomatic gallstone disease but had no significant comorbidities or medications known to affect glucose or lipid  metabolism. | Patients were  randomized and treated with either placebo or the FXR agonist OCA at 25mg/day for 3 weeks (placebo n=6, OCA n=5). | FXR activation reduced hepatic TG, reducing the expression of FFA and TG synthesis genes. | Clifford, BL., et al. 146 |
| Double-blind placebo- controlled phase  2 trial. 140  patients with noncirrhotic NASH. | NASH patients randomized received ciclofexor 100 mg, 30 mg or placebo orally once daily for 24 weeks. | Improvement of liver steatosis, and  improved liver biochemistry markers. Side effects: moderate to severe pruritus associated mainly to 100 mg cicloflexor. | Patel, K., et al. 147 |
| Randomized placebo- controlled study. | NASH patients were randomized to receive  wither 80 mg, 50 mg of MET409 (FXR non- | MET409 lowered liver fat content, induced  ALT reductions (in 30- 50% of patients), | Harrison, SA., et al. 148 |

| 58 patients with NASH. | bile acid agonist) or placebo. | decreases high-density lipoprotein cholesterol and increased low- density lipoprotein cholesterol. Side effects: pruritus. |  |
| --- | --- | --- | --- |

**Suppl. Table 4.** Microbiome modulation intervention studies in CLD: Probiotic administration.

| **Animal studies** | | | |
| --- | --- | --- | --- |
| Population | Characteristics of the intervention | Results | Ref. |
| Forty male C57BL/6N mice were treated with probiotic and/or Salvia miltiorrhiza. | Probiotic candidate strains were administered to mice fed with HFD diet with or without  combination of Salvia miltiorrhiza polysaccharide. | The combination of probiotics with Salvia miltiorrhiza improved insulin resistance, liver serum parameters, and decreased TG and cholesterol in serum in MASLD mice induced by HFD | Wang, W., et al. 154 |
| Male Sprague Dawley rats | HFD fed rats were fed with HFD for 6 weeks. Metformin alone or in combination  with *Lactobacillus reuteri* DSM 17938 (*L. reuteri*) were given orally for 4 weeks; meanwhile, metronidazole (15 mg/kg/day, p.o.) was administered for 1 week. | Probiotics in combination with  metronidazole and metformin significantly ammeliored IR, lipid profile, liver function, oxidative stress, inflammatory and autophagic markers than using each treatment alone.  Besides, this combination resulted in disappearance of steatosis, inflammation and vacuolation within hepatic architecture. Moreover, it normalized short chain fatty acids (SCFAs) as well as *Firmicutes and Bacteroidetes* faecal  contents | Seif el-Din, SH., et al. 155 |
| **Human studies** | | | |
| Population | Characteristics of the intervention | Results | Ref. |

| A meta-analysis of 11 randomized controlled trials reporting the  effect of  probiotics, prebiotics, or symbiotics (2020-  2022) and encompassing 741 participants. | Effects of probiotics, prebiotics, or  symbiotics were evaluated in MASLD patients and control subjects. | Probiotics, prebiotics, and symbiotics supplementation can potentially improve liver enzymes, lipid profiles, and liver steatosis in patients with MASLD. No significant effects were found in hepatic fibrosis. obesity and MS features. | Xing, W., et al. 153 |
| --- | --- | --- | --- |
| A randomized double-blind placebo- controlled trial involving ultrasound- diagnosed MASLD patients (*n* = 39) | Patients were supplemented with either a probiotic (MCP® BCMC® strain s) or a placebo for a total of 6 months. Multi-strain probiotics (MCP®BCMC® strains  ) containing six different *Lactobacillus* and *Bifidobacterium* species at a concentration of 30 billion CFU were  used. | The use of probiotics did not result in any significant improvement in MASLD patients. However, at the microenvironment of the small intestine probiotics stabilized mucosal immune function and gut permeability. | Mohamad Nor, MH., et al. 156 |

**Suppl. Table 5.** Microbiome modulation intervention studies in CLD: FMT.

| **Animal studies** | | | |
| --- | --- | --- | --- |
| Population | Characteristics of the intervention | Results | Ref. |
| Forty-two male 5- week-old C57BL6/J mice | Mice were  randomized into 1.  control, 2. HFD for 18  weeks, 3 caloric  restriction (12 weeks on HFD and 6 weeks under caloric restriction), 4. FMT (HFD plus FMT at weeks 17 and 18). | FMT, and, especially, FTM with own feces (collected before developing obesity) potentiated the effects of a moderate calory restrictions on weight loss and adiposity, by decreasing feed  efficiency and  increasing adipose  tissue lipolysis. Although some  changes were identified in bacterial richness only significant increases in  *Bifidobacterium* and | Pérez- Matute, P., et al. 162 |

|  |  | *Blautia* genera were observed, suggesting that other mechanisms different from bacterial microbiota engraftment participates in these beneficial effects. |  |
| --- | --- | --- | --- |
| 5-week-old male C57BL/6J Narl mice (n=49). | 12-week-HFD-fed animals received FMT gavage from control- fed exercised animals or from HFD-fed exercised animals, 5 times per week for a period of 12 weeks. | FMT from control-fed exercised animals improved metabolic profiles of sedentary- HFD animals. The transmissible beneficial effects of FMT were associated with bacterial  genera *Helicobacter*, *O doribacter* and AF12 and overexpression of oxidative phosphorylation and glycolysis genes | Lai, ZL., et al.  163 |
| Specific pathogen free male  C57BL/6 mice (n=36). | 8-week-HFD-fed Animals were fed with HFD for 8 weeks. After an 8-week HFD, FMT treatment was initiated and carried out for 8 weeks. | FMT improved gut microbiota composition with elevated abundances of the beneficial  bacteria *Christensenell aceae* and *Lactobacillu s*, it also elevated butyrate concentrations of cecal content and increased intestinal TJ protein ZO-1, resulting in relief of endotoxima. Steatohepatitis was alleviated in terms of lipid accumulation and  inflammation. | Zhou, D., et al. 164 |
| Male Sprague- Dawley rats weighting 180 ±  20 g. | Rats were exposed to different stressors for four weeks and underwent to FMT from healthy controls by gavage for 14 consecutive days. | FMT modified gut microbiota by increasing  Firmicutes and decreased *Bacteroidet es* and *Desulfobacterot a* at phylum levels. FMT also reduced the  loss of villi and epithelial cells; | Rao, J., et al.  171 |

|  |  | suppressed the  inflammatory cell infiltration in the ileum; increased the expression of ZO-1, occluding and protected the mucosal layer function. |  |
| --- | --- | --- | --- |
| **Human studies** | | | |
| Population | Characteristics of the intervention | Results | Ref. |
| Randomized, double-masked, placebo- controlled trial.  87 adolescents with obesity  (BMI ≥ 30 kg/m2) | Single course of oral encapsulated fecal microbiome from 4 healthy lean donors of the same sex or saline placebo was administered. | No significant effects on weight, although a reduction in abdominal adiposity was observed. After a 26- week-period of follow up, changes in the overall gut microbiome composition and a resolution of the MS were identified  especially among female participants. | Leong KSW., et al. 167 |
| Double-blind randomized placebo- controlled pilot trial. 24 adults with obesity and mild-moderate IR. | randomized adults received oral FMT capsules from healthy lean donors for 6 weeks. | Despite engraftment, no  significant differences between groups in most glycaemic outcomes, weight, or body composition over a 12-week periodwere identified. | Yu, EW., et al. 168 |
| Randomized and controlled clinical trial with 75 MASLD patients | Individuals in the non- FMT group were given probiotics orally. In the FMT group, patients were randomized to receive FMT with donor stool (heterologous) *via* col onoscopy, followed by three enemas over 3 days. Both groups were also required to maintain a healthy diet and keep regular  exercise for more than 40 min every day | One month after treatment FMT slightly reduced fat  accumulation in the liver. FMT had better effects on gut microbiota reconstructionin lean MASLD rather than obese MASLD patients. | Xue, L., et al.  169 |

# SUPPLEMENTARY MATERIAL AND METHODS

## Maintenance of mice and animal experimentation

All animal experiments were designed and performed in accordance with the Spanish laws and regulations on animal protection (PROEX 264.2/23). Mice were kept in a specific pathogen-free facility of the School of Biology (Complutense University of Madrid) in a temperature-controlled room with 12-hour light/dark cycles, with free access to food and water, according to the guidelines of the Federation for Laboratory Animal Science Associations (FELASA). 20-week-male mice in a C57BL/6J background from Janvier Labs were used.

## Mice sacrifice, gut extraction and colon processing

Mice were sacrificed by an overdose of isofluorane (Solvet), inhalation. The abdominal cavity was cut open along the *Linea alba*, two relaxation cuts were placed along the costal arches at the lateral abdominal walls. The total gut was extracted, and the intestinal content was carefully poured out by using a syringe with PBS. Colon was separated and proximal part was placed into a plastic cassette and fixed in Carnoy solution (60% methanol, 10% acetic acid, 30% chloroform). 24-48h later the cassettes were dehydrated and embedded in paraffin WAX. After that the paraffin blocks were cut in 5μm sections using a rotatory microtome RM2125 RTS (Leica Biosystems, Germany).

## Immunofluorescence staining

5 μm colon sections were rehydrated by rinsing in Xylol and decreasing concentrations of ethanol (100%, 95%, 70%) and water. Antigen demasking was performed by boiling the sections in 10 mM sodium citrate buffer, pH 6.0. To prevent non-specific binding, sections were blocked with 5% goat serum in PBS for 1 h. The slides were exposed overnight to primary antibody (Mucin-2, Santa Cruz; USA, sc-15334, 1:800 / Zo-1, Invitrogen; USA, 61-7300, 1:100) at 4ºC. The next day, samples were rinsed in PBS for washing and incubated with secondary antibody (Goat-Anti-Rabbit Alexa Fluor 488; 1:500; A11001, Invitrogen; USA) for 1 h. After that samples were washed and Vectashield mounting medium containing DAPI (Vector Laboratories, UK) was used for counterstain and mounting. Pictures were taken in a Leica AF6000LX with Leica

type microscope DMI6000B together with LAS_X_Core software (Leica Biosystems, Germany) at 20x magnification.

## Hematoxylin and eosin staining (H&E)

H&E staining was performed in 5 μm colon embedded paraffin sections. Deparaffinization and rehydration were performed through changes in decreasing ethanol concentrations (100%, 95% and 70%) and water. Nuclei were stained in blue by rinsing the slides in hematoxylin (AppliChem, Germany). Cytoplasm and interstitial areas were stained in eosin (Sigma-Aldrich, USA) and further washed in water. Samples were then dehydrated by using increasing concentrations of ethanol (95%, 100%), xylene, mounted with Roti-Histokitt (Carl Roth, Germany) and covered by a coverslip. Photomicrographs were taken through an optical microscope (Nikon, Japan) at 10x and 20x magnification.
